# Sterilization by Cooling in Isochoric Conditions: The Case of *Escherichia coli*

**DOI:** 10.1371/journal.pone.0140882

**Published:** 2015-10-19

**Authors:** Samuel Salinas-Almaguer, Abril Angulo-Sherman, Francisco Javier Sierra-Valdez, Hilda Mercado-Uribe

**Affiliations:** CINVESTAV-Monterrey, Apodaca, Nuevo León, México; University of California at Berkeley, UNITED STATES

## Abstract

High hydrostatic pressure (HHP) affects the structure, metabolism and survival of micro-organisms including bacteria. For this reason HHP is a promising treatment in the food industry. The aim of this work is to evaluate the effect of high pressure, under isochoric cooling conditions, on *Escherichia coli*, where such high pressure develops due to the fact water cannot expand. We combine survival curves obtained by spectrophotometry and images of atomic force microscopy in this study. Our results show that cooling at -20 and -30°C leads to a partial destruction of a *Escherichia coli* population. However, cooling at -15°C causes a total extermination of bacteria. This intriguing result is explained by the phase diagram of water. In the first case, the simultaneous formation of ice III and ice Ih crystals provides a safe environment for bacteria. In the second case (-15°C) *Escherichia coli* remains in a metastable and amorphous free-of-crystals liquid subjected to high pressure. Our work is the first experimental study carried out to inactivate *Escherichia coli* under isochoric cooling conditions. Unlike HHP, which is based on the application of an external load to augment the pressure, this technique only requires cooling. The method could be used for annihilation of other *Escherichia coli* strains and perhaps other micro-organisms.

## Introduction

Microbial inactivation has been the subject of numerous studies due to the fact micro-organisms are the main responsible for food spoilage and diseases. Bacterial species are part of these organisms and one of the most extensively studied is *E. coli*. The identification of cellular targets and mechanisms that induce the *E. coli* death are key questions that have not been completely resolved. In order to elucidate these queries, different methods have been developed and their limitations explored. The most used are: ionizing radiation, pulsed high electric fields, plasma formation and high hydrostatic pressure. All of them have the advantage that they are less harmful than thermal techniques.

Irradiation procedures involve the application of electromagnetic waves (generally, *γ* beams) on the sample. Ionization essentially causes oxidative and chromosomal damage, as well as the loss of repair mechanisms. Although some authors have suggested that these effects are the origin of cell death, it is not clear how decisive they are [1–3]. Recently, several studies have reported bacterial inactivation in liquids based on a plasma created by discharges generated from high voltages or currents. A sterilization phenomenon is also investigated with a pulsed electric field method, which generates pores in the membrane and organelles. Both techniques have not shown a relationship between the damage observed and microbial inactivation [1, 4–7].

High hydrostatic pressure (HHP) processing is considered as one of the most promising techniques for several reasons. Although HHP causes some effects on nutritional qualities and sensory attributes of food products (such as meat discolouration, inhibition in enzyme activity and protein denaturation), and these effects are small, HHP has become an attractive alternative for food, tissues and organs preservation [8, 9]. Additionally, HHP has shown to be a bacterial growth inhibition method [10–14]. For instance, Suppes et al. [15] suggested that high pressure does not directly kill *E. coli* but rather triggers a sequence of events that ends with cell death. There is a documented evidence that HHP affects several structures and metabolism of *E. coli*. In this context, damage in cellular membrane has been observed by two different ways: the leakage of ATP or proteins and the loss of osmotic responsiveness (using a fluorescent dye propidium iodide, that usually do not permeate the membranes of healthy cells). Also, using transmission electron microscopy (TEM), denaturation of proteins and DNA have been exhibited through the presence of amorphous compacted and enlarged fibrillar regions. Finally, damage in ribosome, oxidative stress and disturbances on the recovery mechanisms have been reported [1, 14, 16–19]. Such injuries depend on the magnitude of applied pressure, size and resistance of the strain, as well as the phase of the cell at the time of exposure to pressure. In turn, pressure resistance depends on the strains: species with more fluid membranes are more resistant [20, 21].

All in all, there is a controversy about the mechanisms leading to the loss of viability by high hydrostatic pressure in several bacterial species. Membrane damage seems to be the strongest cause for cell death due to the fact biological activities are highly dependent on the membrane integrity [1, 11, 12, 16, 17, 21, 22].

In the present study, we have explored the effect of an isochoric cooling process on *E. coli* cultures. Unlike high hydrostatic pressure (HHP), where there is an external mechanism to apply a load and therefore augment the pressure, our technique only requires cooling. The volume does not change when cooling the sample below the freezing point. In this way, the pressure inside the container increases substantially as indicated by the phase diagram of water.

An isochoric cooling process has been also proposed in interesting reports as an alternative method to achieve cryopreservation even with more advantages than isobaric processes. This probably leads to chemical damage through the increase of ions in cell during freezing [23–25].

## Materials and Methods

### Culture media and growth conditions

*E. coli* MG-1655 was used in this study. 200 *μ*L of a bacteria aliquot previously stored at -20°C in a Luria Bertani (LB) broth medium with 30% of glycerol were added into a volume of 20 mL of LB medium and left for incubation during 24 h at 37°C. For a control sample we add one part of this incubated culture (200 *μ*L) to a 2.4 mL of LB medium. The rest of the culture was subjected to the cooling process (detailed below). Four wells of a flat-bottomed 96-well microplate were filled with 200 *μ*L of the previous suspension and sealed with 50 *μ*L of mineral oil to prevent evaporation. Afterwards, growth curves were obtained using a spectrophotometer (Multiskan GO Thermo Scientific). The curves were averaged and used as controls. This step was carried out using a wavelength of 600 nm and a temperature of 37°C. The suspensions were continuously stirred during the measurements.

### Cooling process

As above mentioned, part of the previous culture was distributed into two containers. The containers were stainless-steel cylinders 3.2 cm long and 4.7 cm in diameter. Each cylinder contained a central cavity 1.1 cm in diameter and 2.44 cm deep. The containers were sealed with a 0.95-cm-wide stainless steel disk. The disks have a central knob 0.2 cm thick surrounded by an O-ring allowing the knob to fit tightly inside the cylinder and ensuring a hermetic seal. The disks were held with six screws. The first container was completely filled with 2.13 mL of the *E. coli* suspension, the other one was filled to 90% of this volume. Both of them were placed inside a thermal bath during 20 h at a fixed temperature. We carried out experiments using three different cooling temperatures: -15, -20 and -30°C. Isochoric conditions were kept in the first (because in this case the volume was fully occupied), while ice Ih formation was possible in the second container because it was not completely filled. The experiments were carried out four times. Due to the fact that Black et al. have reported different biophysical effects on *E. coli* exposed to a wide range of hydrostatic pressures with lethal consequences around 100–200 MPa [22], we expected differences after the cooling process for reasons that will be clarified later.

### Measurement of growth culture

After 20 h the containers were placed in a thermal bath during 1.5 h at 37°C. Subsequently, 200 *μ*L of the cooled samples were poured into a flat-bottomed 96-well microplate to obtain the replication curve (as we explained previously) and compare with those of the control sample.

### Preparation of samples for Atomic Force Microscopy

To evaluate the effect of isochoric cooling on *E. coli*, the samples were analyzed by atomic force microscopy (AFM). Previously, they were washed three times with ultrapure water (1000 mL for 5 min). In order to image the bacteria with AFM, they must be bound to a glass surface. Polyethylenimine (PEI, 750 000 Da) was used to create a positively charged glass surface that promoted irreversible adhesion of bacteria. To prepare these slides, the PEI solution (0.2%, 1 mL) was placed on the glass slide for 3–5 h and then rinsed with MilliQ water. A bacteria suspension (1 mL) was placed on the glass slide and after 30 min the bacteria-coated slide was rinsed vigorously in a stream of deionized water, and dried with nitrogen for imaging in air once the process is finished [26].

### Atomic Force Microscopy

Imaging was performed with an atomic force microscope (Innova Bruker, Santa Barbara, CA) equipped with a small area scanner (maxima scan area of 5 *μ*m x 5 *μ*m) in air at room temperature (25°C). Images were acquired in tapping mode using antimony doped Si cantilevers (Bruker, Santa Barbara, CA) with nominal tip radius of curvature of 2 nm, nominal spring constant of 40 N/m, and nominal resonant frequency of 300 Hz. Experiments were recorded with settings of 512 pixels/line, scanning rate of 0.5 Hz, scan angle of 90° and the minimum force possible. The raw AFM data were flattened and plane-fitted using the Gwyddion 2.38 software package. Three bacteria were analyzed for each experiment.

## Results

### *E. coli* replication

**Fig 1:**
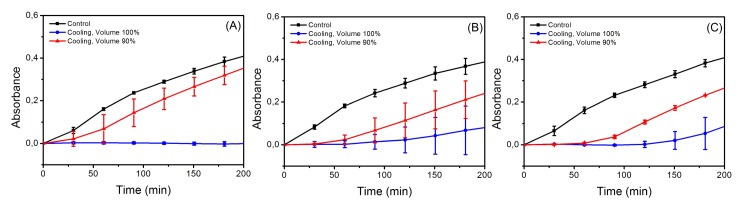
Replication curve for *E. coli* after the cooling process. The samples were subjected to three different cooling temperatures: (A) -15°C, (B) -20°C and C) -30°C. In all the cases the container was filled up to 90% and 100% with the bacteria suspension. The results are compared to the control. Each point corresponds to the mean of four independent experiments.

Fig 1 shows absorbance measurements as a function of time. This illustrates the replication curves for *E. coli* during 180 min after cooling at -15, -20 and -30°C (from left to right). One can observe that in the case of the sample that totally fills the container (blue lines), there is a noticeable contrast between the results at -15°C and the others two temperatures. Indeed, the replication rates and final populations are higher for -20 and -30°C, while at -15°C *E. coli* does not replicate. The red lines correspond to the experiments with the container filled to 90%. In all these cases, replication is observed.

### AFM analysis of *E. coli* after cooling process

**Fig 2:**
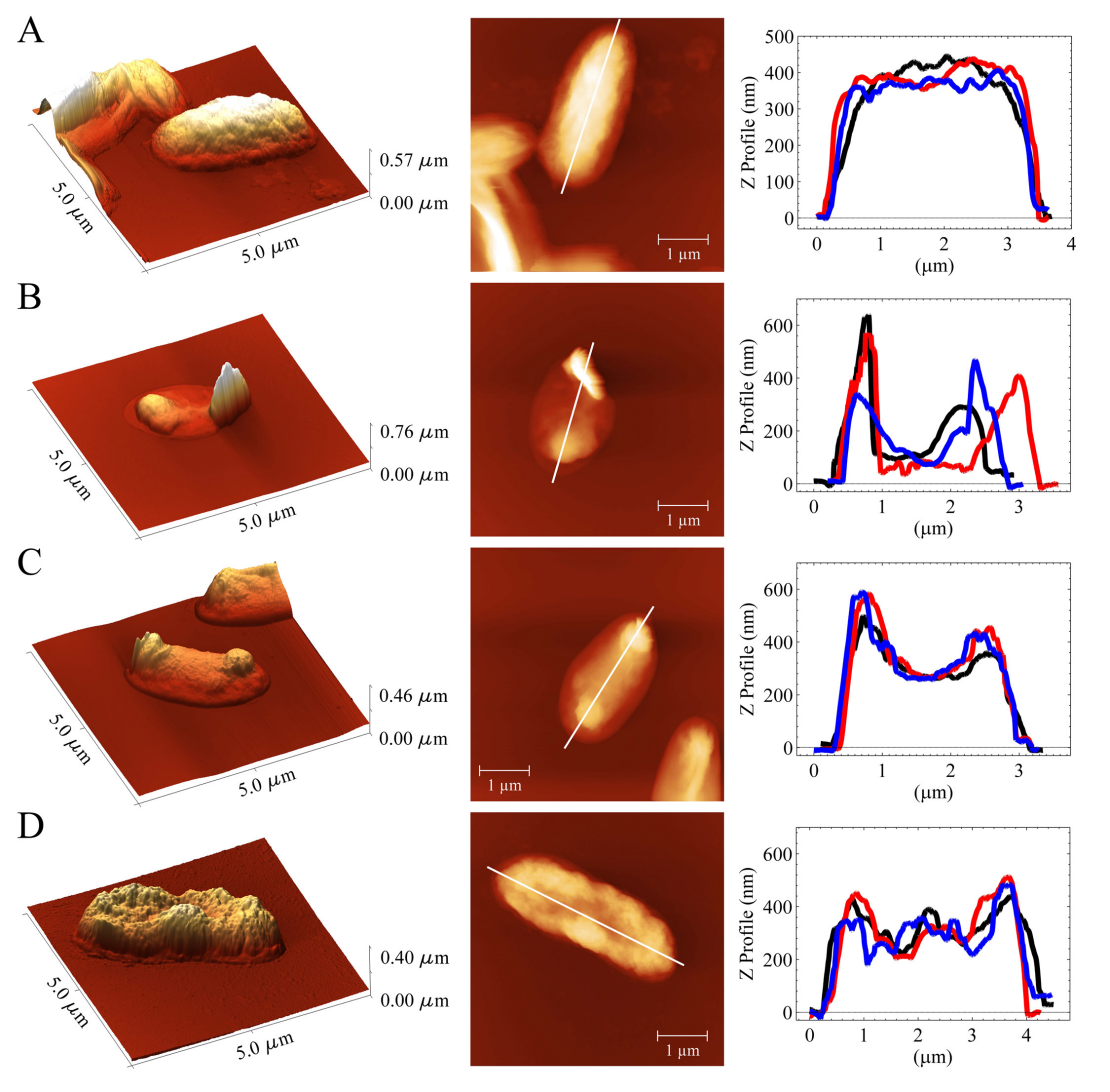
Representative AFM images for treated and untreated *E. coli* samples. (A) Control cells, (B), (C) and (D) cells subjected to cooling process at -15, -20 and -30°C, respectively. For these cases, the container was completely filled with the sample. The first and second column correspond to 3D and 2D topographic images, respectively for only one cell. The third column shows the line profiles for three different cells. Line profiles were taken along the horizontal white line in the 2D representation. Different shades and light intensities in the images indicate different heights: the lighter shades correspond to larger heights.

Fig 2 displays representative images and line profiles obtained by AFM of *E. coli* in the case where the suspension occupied the total volume of the container (note that we plotted three line profiles from different bacteria). There are clear differences between the control sample (A) and those under the isochoric cooling process at: (B) -15, (C) -20 and (D) -30°C. These results reveal that isochoric cooling leads to alterations in the physical integrity of *E. coli*. The most evident alterations involve the membrane damage, changes of the shape and cellular dimensions, exhibition of blister-like protrusions, and rupture of membrane and expulsion of intracellular material (the two last effects are particularly exhibited at -15°C). Note that only at this temperature an unexpected variability in length is obtained and the type of the deformation is even more dramatic. These findings are in agreement with previous studies in cells subjected to elevated hydrostatic pressures, which are achieved by compression [14, 27]. Although no molecular changes are visibly observed in our images, it is possible that ribosomes dissociate, and, consequently, proteins synthesis is inhibited and denaturation of DNA occurs. Indeed, these effects have been already reported around 60 MPa in cultures of *E. coli* [18, 28, 29].

**Fig 3:**
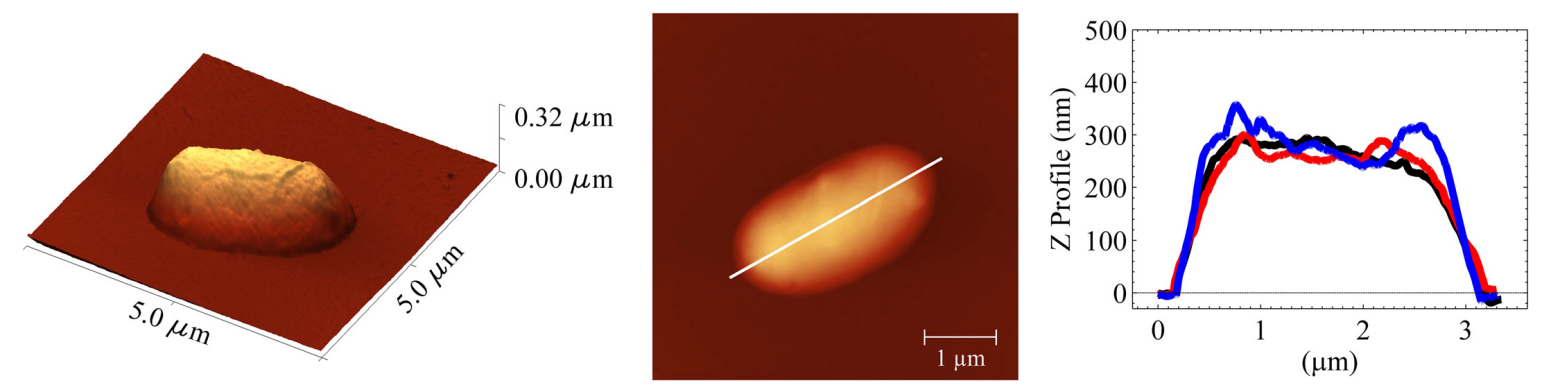
Representative AFM images for treated *E. coli* samples. Cells subjected to cooling process at -15°C. The sample occupied 90% of the container volume. The first and second column correspond to 3D and 2D topographic images, respectively for only one cell. The third column shows the line profiles for three different cells. Line profiles were taken along the horizontal white line in the 2D representation. Different shades and light intensities in the images indicate different heights: the lighter shades correspond to larger heights.

Fig 3 exhibits the effect of cooling at -15°C in the *E. coli* sample which fills 90% of the container. It can be observed that the cell morphology and line profile are similar to the control. This is in agreement with the replication curves shown in Fig 1 (here, only the images for this temperature are displayed because is the most representative case).

## Discussion

**Fig 4:**
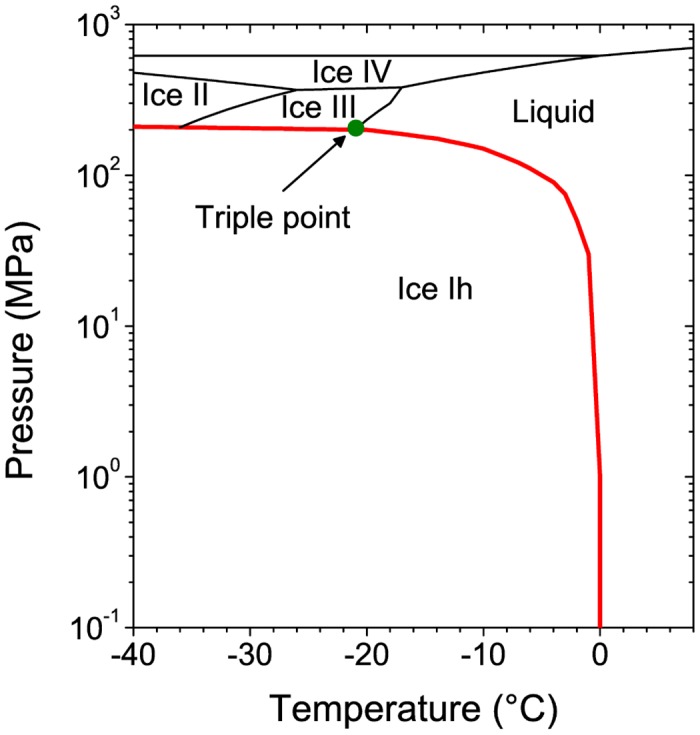
A section of the phase diagram of water. Liquid, crystalline states, and metastability borderlines are shown. The red line represents the path followed by a cooled sample under isochoric conditions.

Recently, we have studied the dielectric properties of pure water in a constrained macroscopic volume (isochoric conditions) as the temperature decreased from 6 to -23°C in steps of 2°C [30]. Under these conditions water cannot crystallize. Based on the phase diagram of water (see Fig 4), as the liquid inside the container is cooled below 0°C, the thermodynamic path follows the coexistence line (in red), where the pressure reaches very high values. Indeed, according to the Clausius-Clapeyron equation [31], $\frac{dP}{dT}=\frac{\Delta H}{T\Delta V}$, where P, T, H, and V are pressure, temperature, enthalpy, and volume, respectively, the pressure increases considerably because the volume is not allowed to change (Δ*V* = 0). The phase diagram indicates that water is liquid until approximately 210 MPa (at -24°C), when the triple point is reached. Since the volume and mass are constant, the density cannot change. At the triple point, a shell of ice III begins to nucleate on the surfaces of the container due to the high pressure pushing against them. Because ice III is denser than water [32], its formation immediately creates space for a thin shell of ice Ih to nucleate. Another interesting finding in our study was that water shows a similar cooperative behavior than ice Ih. In other words, the overall molecular motion of water under isochoric cooling becomes as slow as ice Ih. This effect is produced due to the high pressures inside the container. Although it is not clear what is the phase before the triple point, our results indicate that it is a very viscous liquid [30]. The previous discussion will be important in our next arguments about bacteria replication.

The effect of cooling at -15 -20 and -30°C on the *E. coli* sample filling 90% of the container is simple crystallization. According to the Chatelier’s principle, if the equilibrium conditions (v.gr. temperature and concentration) in a system (bacteria-solvent) are disturbed; then, the system readjusts itself to counteract this effect. Due to the osmotic reset at these conditions, cell death is reached. However, our results indicate that there is not a total extermination of bacteria. One can observe that they still replicate (see Fig 1). More interesting results are obtained at isochoric conditions. The replication curves (blue lines) indicate that the effect of cooling is lethal at -15°C. These findings are gripping and completely unexpected. The explanation is based on the phase diagram of water: at -15°C the bacteria remain in a metastable environment (described by the red coexistence line in Fig 4) where no ice is formed and the liquid exerts a pressure able to cause a complete extermination of bacteria. Indeed, this can be confirmed by looking at our AFM images in Fig 2B. In contrast, cooling at lower temperatures (-20 and -30°C) leads only to a partial destruction of the *E. coli* population (red lines in Figs 1, 2C and 2D). The reason is that at such temperatures ice III begins to form, and consequently, ice Ih forms too. At these temperatures, some of *E. coli* population dies, and other bacteria are sheltered inside the ice crystals (the reader should recall that ice does not fully exterminate bacteria as observed in the red lines of Fig 1). So, after the cooling process surviving bacteria start to replicate.

HHP is a method commonly used in the food industry to retain the texture and colour of a product, while inhibiting micro-organisms [21, 33, 34]. Since cooling in isochoric conditions reaches the same pressure than HHP, we would expect to retain these features too. However, the main advantage of the present method over HHP is that a sophisticated equipment using a pressure pump to generate high pressures is not needed; only a container capable of supporting high pressures when cooling is required.

In summary, we investigated the effect of cooling on the survival of *E. coli* (MG-1655) cultures inside a stainless-steel container that endures internal pressures developed during cooling in isochoric conditions. Survival curves analysis and AFM images showed that when the expansion is not permitted, the effect on *E. coli* depends on the pressure and temperature inside the container. The simultaneous formation of ice III and Ih crystals, which form close to the triple point, provides a safe environment for *E. coli* that prevents the total extermination of bacteria at temperatures around the triple point of water. In contrast, at -15°C, the bacteria in the suspension is in an amorphous liquid (with no ice crystals subjected to high pressures). Since bacteria do not have a medium where to protect, the result is their total extermination. These findings are puzzling because one would expect that as the temperature decreases the damage would be greater. Future work will explore the effect of temperature conditions between 0 and -15°C.

The present method could be used to guarantee sterile conditions for other type of samples, depending on the characteristics of microbial species and medium composition. Due to the fact some strains of bacteria could be more resistant than others for a given pressure, additional experiments are needed prior to the application of the technique discussed here for food industry.
